# Paying Patients

**Published:** 1906-03-31

**Authors:** 


### PAYING PATIENTS.

### SCHEME FOR A HOSPITAL INSURANCE POLICY WITH CO-OPERATION.

The Duke of Northumberland presided on March 23 ultimo at the twenty-eighth annual meeting of the Home Hospitals Association for paying patients, Fitzroy House, Fitzroy Square. The report showed that during 1905, 418 patients were admitted out of 494 who applied. Of the refusals 35 were for want of room, 10 cases were ineligible, in 11 the rate of payment was too high, while 20 were for other reasons. The receipts for the year were £8,451 and the expenditure £7,981. In the 30 beds available the average number of patients was 23, the average stay of each patient being 19 days. A new operating table of the most improved kind was installed during the year.

In moving the adoption of the report and accounts the Chairman expressed the pleasure he felt at the success and progress of the Society. They had a balance to credit of £470, which must be looked upon as extremely satisfactory on the working of the year, especially as they had paid £500 the first instalment of the repayment of their mortgage. If it had not been for that and the interest they would have had a profit of £1,100 on the year. He thought that showed that, in spite of their liabilities, they were at least solvent, and so long as the institution maintained its high level of efficiency they might certainly hope for continued financial success in the working. The financial side was not, however, the most interesting. They were doing good work in filling a void in one of the philanthropic needs of the social position. Perhaps philanthropic was hardly the word, because philanthropy was not carried on on a commercial basis, and that Association had all along been carried on on a commercial basis, so that those who used it were not indebted to anyone for the benefits they received. And not only was the institution filling a great want, but it was an educational force as well. There was growing up a strong feeling that more might be done in the way of inducing those who received the benefits of hospital treatment to do more to meet the expenses from which they derived so much good. One phase of the educative aspect of the institution was referred to in the clause of the report which spoke of the eighty medical practitioners who had had patients in the hospital during the year. He was sure that through them the great principle they had at heart would be made known throughout the length and breadth of the land. In conclusion, he thought they might congratulate themselves on having secured the services of Mr. Gavin Hamilton as Secretary. (Hear, hear.)

#### Not a New Question.

Sir Henry Burdett seconded the resolution. He said it was very interesting, in looking back over the history of that institution, to realise the conservatism of the British people. There had lately been a correspondence in the Press on the need for the establishment of a pay hospital near the residences of the consultants, to which patients could go who could pay a relatively small sum for what they needed. This had carried him back to 1877, when they were establishing that Association. He found that the present discussion followed nearly the identical lines of that of thirty years ago. At that time the *Lancet*, in a leading article, described the idea of a pay-hospital as "not by any means new."

### The Death of Hospitalism.

Since that time conditions had greatly altered. It might be interesting to recall that at the Mansion House meeting on June 27, 1877, at which that Association took its birth, a member of the *Lancet* staff said he believed it was the opinion of the great mass of professional men that it was desirable to reduce the number of hospitals for surgical operations to a minimum, for every gentleman of standing would sooner treat an amputation or any such case at a private house. There was then some reason for that attitude, for in the four leading Metropolitan hospitals, containing upwards of 1,800 beds, Professor Erichsen in a paper on "Hospitalism" had shown the mortality after major operations to have been 37.8 per cent. No doubt the speaker had that in view when he stood up and resisted the idea of that Association on account of the dangers of "hospitalism." But all that was changed to-day, and it was curious to notice that the *Lancet* of the previous week had said: There are many operations which no surgeon would undertake in an ordinary private house. The great change in opinion and practice thus revealed they owed to the life-work of Lord Lister and the eminent surgeons who now performed myriads of operations which then no one dare attempt, and it was interesting to note that it was mainly with surgical cases that that Association dealt and in which it had achieved such success and been of such invaluable aid to practitioners and patients.

### Paying Wards and Finance.

At the Mansion House meeting in 1877 suggestions were made for a pay-hospital and also for the provision of paying wards in connection with the voluntary hospitals, and one of the first results of their efforts was that the committee were approached by the Treasurer of St. Thomas's and a tentative offer was made with a view to their utilising one of the pavilions at St. Thomas's as a home hospital. The suggestion was that they should have 69 beds, of which 50 on an average would be occupied. Of these 30 could be at the disposal of the public at £2 a week. The reason the Home Hospitals Association did not accept the offer of St. Thomas's was on financial grounds, that the margin on the suggested rates of payment was only £400 a year, with a much larger rental to be paid. It was not thought right to attempt such an experiment under those circumstances, and in a large pavilion costing £40,000. Indeed, the financial question had always been the difficulty in regard to the establishment of pay-hospitals all over the world. They started at Fitzroy House in 1880, and at the end of the first year found that they had been successful on the lines laid down, that they must be self-supporting. The committee had always held that their results must show a margin of profit. They had been going on for twenty-six years, and had continued to maintain the hospital on that basis. Four years after that opening of Fitzroy House, the London Hospital had promoted a special Act of Parliament, in which they obtained power to take paying patients not exceeding in number 10 per cent. of the available beds. But as a matter of fact no paying patients had ever been taken at the London Hospital, and he did not think they ever would be; the reason being the situation of the hospital and the requirements of the poor. At Guy's they had a paying branch where the public could get accommodation for three guineas a week. At St. Thomas's a pay-pavilion had been working for many years, known as St. Thomas's Home, where the charge was from three guineas upwards. At St. Thomas's, too, they had taken a further step by allowing poorer people, who could yet afford to pay something, to have accommodation in the ordinary wards for one guinea a week. Nursing homes, too, had increased and improved; but so far as providing accommodation for the lower middle classes at about two guineas a week, no progress had been made. As he had said, the cause was financial. Up to four years ago they had a few beds at three guineas, but they found in practice that that sum was too low to cover the inclusive charges, so when the hospital was rebuilt they determined to increase the minimum charge to four guineas a week, and at that it had remained. If they looked at the accounts of the larger hospitals they would see that the cost of treating patients in the wards averaged about two guineas a week.

### Continental and United States Methods.

It had been said that on the Continent accommodation was provided for the middle-middle and lower-middle classes in separate pay-hospitals, but that was a misapprehension. In several countries there were three or four grades of patients, and each patient paid something. The cheapest grade was for the poor, who were paid for by the commune or municipality. The next rate was rather more, though not necessarily remunerative; then came a rate which was inclusive, and represented the cost of the treatment received, or rather more; while the highest grade yielded a profit on the fees charged. But with Government or municipal aid the problem became simple, and that system had the advantage that no one need forfeit self-respect by receiving free treatment. In the United States most of the large hospitals had pay wings or pavilions, but it was found that the cost of treating hospital patients in the way they had to be treated in paying wards was much higher than from two to three guineas a week. The cost ranged from £5 to £15 a week, and what he learned in America confirmed their early experience at that hospital. The American public, like the English, would not pay unless they could get a private room. The accommodation at the lowest rate (£5) was very limited; in a large institution like the Roosevelt Hospital, containing 46 private wards, there were only two where the rates were as low as £5 a week. Some American hospitals were supported entirely by the municipality, like the Boston City Hospital, one of the best organised and administered. There the income from paying patients in 1904 was rather under £14,000, of which £5,000 was paid by the State and £1,300 by different cities and towns, for patients sent in by them, showing that the Continental system was partially followed there. On the Continent, in the United States, and in the Colonies there were a large number of efficient pay-hospitals run by private individuals, where the rates were often higher than those quoted for the Roosevelt Hospital.

### The Financial Problem.

They were brought back, therefore, to the financial problem, and unless that were solved they would never get what they wished to obtain—a bridge between a paying hospital like that one and the voluntary or free hospital, namely, the paying ward. The real solution to the question was to be found in co-operation. Co-operation between the hospitals and the public, and among the hospitals themselves, which, by association, would be able to give the public what was wanted; while the public by a system of insurance would be able to pay the cost of an operation and three guineas a week for hospital care.

Pay-Pavilions, Doctors, Fees, and Insurance.

If, then, in connection with the hospitals that had available sites pay-pavilions could be erected, they would be able to place the pay system on a proper basis. There must be a separate entrance to these pavilions distinct from that to the general hospital, though the pay-wards would be managed and worked by the hospital staff. The paying patients would have the right to select their own practitioner; payments to the doctors would have to be on a reduced scale to be arranged on the Birmingham plan, and there must be some system of pooling the patients' payments or insurance, out of which the doctors' fees would be provided. If this were done it could be shown that adequate accommodation and treatment, with interest on capital and a sinking fund, could be provided for a payment of three guineas a week. As to the question of insurance, they must have a hospital policy on the lines of an accident and sickness policy. He was able to state that actuaries had been working at a suggested scheme for years, and now saw their way to provide the proposed policy giving patients the necessary hospital treatment and some weeks at the seaside for the period of convalescence for an annual premium of little more than the ordinary accident policy. If this were carried out, and the hospitals co-operated, as he knew some at least were willing to do, there was only one difficulty remaining—the provision of the money for the erection and equipment of the first pay-pavilions. Estimates of the probable cost had been prepared, and if some one like the late Mr. Peabody would show enough public spirit to advance the necessary quarter of a million, the rates of pay for patients in those pavilions would yield 4 per cent. on the outlay after paying the other expenses, 3 per cent. of which might be paid in interest and 1 per cent. for a sinking fund to return the capital. If that money were available a system of pay-pavilions could be in practical working within three years, and would soon be self-supporting. He had reason to know that the medical profession would be ready to co-operate, as they had done at Birmingham, by attending the working classes at a reduced fee, in accordance with their circumstances and ability to pay. If they could get some such system started in a practical way it would bring about great good. It would not only be a great comfort and relief to many relatively poor but self-respecting people, but it would cut at the roots of hospital abuse, and would provide that nurses should have an opportunity of better training in private nursing than they could get anywhere in this country in a general hospital. By relieving the pressure on the free beds, the managers of the hospitals would be enabled to show that the actual contributions of subscribers were adequate to their needs, and so at last the voluntary system of hospitals would be placed on a solid and sure foundation. He had always wished that the Home Hospitals Association should be an educational pioneer in this matter as the parent of the pay system, and he hoped that what he had said that afternoon would do something to help forward the movement.

The resolution having been agreed to,

Mr. Bland Sutton proposed a vote of thanks to Miss Pearson and the nursing staff, warmly eulogising their services. Everyone who had to do with them, he said, knew they were thoroughly trained and efficient for private nursing.

A vote of thanks to the Chairman brought the proceedings to a close.

